# Preexisting Japanese Encephalitis Virus Neutralizing Antibodies and Increased Symptomatic Dengue Illness in a School-Based Cohort in Thailand

**DOI:** 10.1371/journal.pntd.0001311

**Published:** 2011-10-04

**Authors:** Kathryn B. Anderson, Robert V. Gibbons, Stephen J. Thomas, Alan L. Rothman, Ananda Nisalak, Ruth L. Berkelman, Daniel H. Libraty, Timothy P. Endy

**Affiliations:** 1 Department of Epidemiology, Rollins School of Public Health, Emory University, Atlanta, Georgia, United States of America; 2 Department of Virology, Armed Forces Research Institute of Medical Sciences, Bangkok, Thailand; 3 University of Massachusetts Medical School, Worcester, Massachusetts, United States of America; Centre for Cellular and Molecular Biology (CCMB), India

## Abstract

**Background:**

Dengue viruses (DENVs) and Japanese encephalitis virus (JEV) have significant cross-reactivity in serological assays; the clinical implications of this remain undefined. An improved understanding of whether and how JEV immunity modulates the clinical outcome of DENV infection is important as large-scale DENV vaccine trials will commence in areas where JEV is co-endemic and/or JEV immunization is routine.

**Methods and Findings:**

The association between preexisting JEV neutralizing antibodies (NAbs) and the clinical severity of DENV infection was evaluated in a prospective school-based cohort in Thailand that captured asymptomatic, non-hospitalized, and hospitalized DENV infections. Covariates considered included age, baseline DENV antibody status, school of attendance, epidemic year, and infecting DENV serotype. 942 children experienced at least one DENV infection between 1998 and 2002, out of 3,687 children who were enrolled for at least one full year. In crude analysis, the presence of JEV NAbs was associated with an increased occurrence of symptomatic versus asymptomatic infection (odds ratio [OR] = 1.55, 95% CI: 1.08–2.23) but not hospitalized illness or dengue hemorrhagic fever (DHF). The association was strongest in children with negative DENV serology (DENV-naive) (OR = 2.75, 95% CI: 1.12–6.72), for whom the presence of JEV NAbs was also associated with a symptomatic illness of longer duration (5.4 days for JEV NAb+ versus 2.6 days for JEV NAb-, p = 0.048). JEV NAbs were associated with increased DHF in younger children with multitypic DENV NAb profiles (OR = 4.05, 95% CI: 1.18 to 13.87). Among those with JEV NAbs, the association with symptomatic illness did not vary by antibody titer.

**Interpretation:**

The prior existence of JEV NAbs was associated with an increased probability of symptomatic as compared to asymptomatic DENV illness. These findings are in contrast to previous studies suggesting an attenuating effect of heterologous flavivirus immunity on DENV disease severity.

## Author Summary

Dengue viruses (DENVs) and Japanese encephalitis virus (JEV) have significant cross-reactivity in serological assays, but the possible clinical implications of this remain poorly understood. Interactions between these flaviviruses are potentially important for public health because wild-type JEV continues to co-circulate with DENV in Southeast Asia, the area with the highest burden of DENV illness, and JEV vaccination coverage in this region is high. In this study, we examined how preexisting JEV neutralizing antibodies (NAbs) influenced the clinical severity of subsequent DENV infection using data from a prospective school-based cohort study in Thailand that captured a wide range of clinical severities, including asymptomatic, non-hospitalized, and hospitalized DENV infections. We found that the prior existence of JEV NAbs was associated with an increased occurrence of symptomatic versus asymptomatic DENV infection. This association was most notable in DENV-naives, in whom the presence of JEV NAbs was also associated with an illness of longer duration. These findings suggest that the issue of heterologous flavivirus immunity and DENV infection merits renewed attention and interest and that DENV vaccine developers might incorporate detailed assessments of preexisting immunity to non-DENV flaviviruses and histories of vaccination against non-DENV flaviviruses in evaluating DENV vaccine safety and efficacy.

## Introduction

The dengue viruses (DENV) and Japanese encephalitis virus (JEV) are closely-related members of the virus family *Flaviviridae*. DENV and JEV co-circulate in the Indian subcontinent and in Southeast Asia, where they are important causes of human disease and mortality. The co-occurrence of JEV and DENV has been documented in Thailand since 1969, when severe epidemics of each were observed in the Chiang Mai valley region [1]. There is no licensed DENV vaccine and vector control efforts have been largely ineffective in containing transmission. While inactivated and live-attenuated JEV vaccines are licensed for use in humans, vaccination does not interrupt the primary JEV transmission cycle involving pigs, waterfowl, and *Culicine* mosquitoes [2]. Despite reported high levels of JEV vaccination (estimated to be 84% in 1998 and 98% in 2008), infections continue to be detected in Thailand each year [3].

JEV and DENV exhibit significant serological cross-reactivity, which can complicate assessment of the relative burdens of each in co-endemic areas and their possible interactions [4], [5]. There exists limited, inconclusive evidence regarding the clinical implications of prior JEV exposure or JEV vaccination and the severity of subsequent DENV infection. Using observed interactions between DENV serotypes as an analogy, JEV/DENV cross-reactive immunity may possibly be protective [6], detrimental [7], or inconsequential. Hoke *et al*. reported that recipients of inactivated JEV vaccine (JEVAX) experienced a non-significant decrease in the occurrence of DHF relative to placebo during the first two years after vaccination and that, among DHF patients, vaccinees experienced milder disease [8]. There was no evidence of an association between JEV vaccination and the occurrence of DHF in a study of hospitalized DENV patients in Bangkok [9]. There has been no reported increase in adverse events following live-attenuated DENV vaccination of JEV-immune volunteers [10], [11]. However, DENV vaccine recipients have demonstrated heightened and broadened DENV antibody responses and antibody responses of longer duration in the setting of preexisting heterologous flavivirus immunity [12]–[14].

Two human studies have provided evidence of a possible protective effect of the opposite sequence; i.e., DENV exposure followed by JEV infection. Burke *et al* found decreased clinical severity in JEV-infected hospitalized patients with higher levels of flavivirus-cross-reactive IgG in Thailand, presumed to be attributable to prior DENV infection [15]. Hammon observed that following the eradication of DENV from Guam in 1945, a subsequent large JEV epidemic in 1947 caused illness in those who were less likely to have been exposed to DENV previously, namely young children and adult expatriates[16], [17].

Animal and in vitro studies of cross-reactivity between various combinations of flaviviruses suggest that the nature of the interactions need not be bidirectional and that the influence of a given virus may vary by serotype and even strain [18]. There exists ample evidence that DENV infection may be enhanced in vitro with heterotypic DENV antibodies [19] and also with antibodies to non-DENV flaviviruses, including JEV [20]. However, a study using sera from JEV-immune Thai individuals found no evidence of enhancement of DENV-2 infection in vitro [21]. Animal studies have suggested a protective role of DENV immunity upon JEV challenge in mice [22] and a protective role of WNV immunity upon subsequent challenge with another member of the JEV antigen complex in a variety of animal models [23]–[26]. In summary, there has been evidence of both protective and detrimental interactions between heterologous flaviviruses; the mechanisms and epidemiological implications of these associations remain unclear.

Potential interactions between flaviviruses are important for public health because wild-type JEV continues to co-circulate with DENV in Southeast Asia, the area with the highest burden of DENV illness, and JEV vaccination coverage in this region is high. As DENV vaccines advance toward licensure and implementation in co-endemic regions, an improved understanding of what constitutes protective immunity with DENV exposure is necessary. Given ambiguous findings from prior studies, we examined how preexisting JEV immunity influenced the clinical severity of subsequent DENV infection using data from a prospective school-based cohort study in Thailand [27], [28]. The availability of information on asymptomatic DENV infections as well as outpatient and hospitalized dengue cases provided a unique opportunity to assess these interactions.

## Methods

### Ethics Statement

The prospective cohort study that generated these data was co-administered by the University of Massachusetts and the Armed Forces Research Institute of Medical Sciences (AFRIMS) for the years 1998–2002. TE, AR, DL, and AN were involved in the design and conduct of the cohort study, for which all subjects provided written informed consent. ST and RG are among the lead scientists presently managing the Department of Virology at AFRIMS, where specimens and data from this study are maintained. The lead author, KA, used only preexisting demographic and laboratory data for the purposes of this retrospective analysis.

The cohort study was approved by the Human Use Review and Regulatory Agency of the Office of the Army Surgeon General, the Institutional Review Board of the University of the Massachusetts Medical School, and the Ethical Review Board of the Ministry of Public Health, Thailand. Secondary data analysis for the purpose of this publication was approved by the Institutional Review Board at Emory University.

### Study Population

Data were collected during a five-year, school-based, prospective cohort study for DENV infections in children in Northern Thailand. The study design and methods have been described previously [27], [28]. Briefly, the study was conducted in Kamphaeng Phet Province from 1998–2002. In January 1998, 2,214 children were recruited from grades 1 through 5 at twelve primary schools. New participants were enrolled from the 1^st^ grade class in January of each year and eligible participants were re-enrolled. The numbers of children enrolled at the start of the active surveillance period each year were 2,044 in 1998; 1,915 in 1999; 2,203 in 2000; 2,011 in 2001; and 1,759 in 2002. A total of 3,687 children were enrolled for at least one year during the study period, with an average of 2.9 years of follow-up per child. At enrollment, the children or their parents were asked about prior JEV vaccination and age of vaccination.

### Study Methods

Serum samples were collected for dengue serology four times each year (January, June, August, and November). All serological and virological testing was performed at AFRIMS in Bangkok, Thailand. Active case surveillance of the participants was conducted from June 1 to November 1, with potential illnesses identified based on absence from school, visit to a school nurse or public health clinic, or admission to the hospital. Absent students were visited by village health workers and evaluated with a symptom questionnaire and an oral temperature. Acute blood samples were obtained for students with a history of fever within 7 days of fever onset, as were 14-day convalescent samples.

### Characterization of Symptomatic Illnesses

Acute and convalescent blood specimens from incident febrile illnesses were tested using immunoglobulin M (IgM) and G (IgG) enzyme immunoassays for DENV and JEV. Acute DENV infections were defined serologically as a DENV-specific IgM level ≥ 40 units and with DENV-IgM > JEV-IgM. The infecting DENV serotype was identified from acute blood specimens using serotype-specific reverse-transcriptase polymerase chain reaction (RT-PCR) or virus isolation. Symptomatic infections were defined as a documented history of febrile illness with virologic or serologic evidence of acute DENV infection. Charts of hospitalized children were independently reviewed and classified as DF or DHF and assigned a severity grade following WHO criteria [29]. If a child experienced a febrile DENV illness but did not meet the criteria for DHF, they were characterized as having DF.

The duration of illness was derived from home visit data and hospitalization records. For non-hospitalized illnesses, the duration of illness was the length of time from the date of school absence or clinic visit to the date of the last home visit at which the child exhibited symptoms consistent with DENV infection. For hospitalized illnesses, the duration of illness was the pre-hospitalization time plus the time in the hospital.

### Characterization of Asymptomatic Infections

Routine specimens were tested for the presence of hemagglutination inhibiting (HI) antibodies against DENV-1 – DENV-4 and JEV using standard methods [30]. The reference virus strains were DENV-1 (Hawaii), DENV-2 (New Guinea ‘C’), DENV-3 (H87), DENV-4 (H241), and JEV (Nakayama). Asymptomatic DENV infections were defined as a four-fold or greater rise in hemagglutination inhibition (HI) titers for any of the four DENV serotypes between two consecutive routine serum samples and without a concurrent four-fold rise in JEV HI titers, or with a concurrent four-fold rise in JEV HI titers but with higher HI titers for any DENV serotype than for JEV.

### Characterization of DENV and JEV Antibody Status

Plaque reduction neutralization titers (PRNTs) were obtained for pre-infection samples using standard methods [31]. Briefly, LLC-MK2 cell monolayers were infected with DENV1 – DENV4 and JEV in the presence of serial dilutions of heat-inactivated patient plasma. The reference virus strains were: DENV-1 (16007), DENV-2 (16681), DENV-3 (16562), DENV-4 (1036), and JEV (Nakayama), for which the sources and passage histories have been previously described [32]. The lowest dilution of serum tested was 1∶10 (corresponding to a final dilution of 1∶20 when combined with an equal volume of DENV); the dilutions and titers reported herein refer to the initial serum dilution. The concentration of patient plasma that resulted in a 50% reduction in plaque formation was calculated using log probit regression. The reciprocal titer of this dilution was defined as the PRNT_50_. A PRNT_50_ <10 was defined as undetectable or ‘negative’ and a titer ≥10 as ‘positive.’ PRNT assays were performed for asymptomatic infections only if the child had not missed school during the observation period. This was done in an attempt to exclude missed symptomatic DENV illnesses that could have been misclassified as asymptomatic seroconversions.

Children were grouped into three categories of DENV immunity based upon their pre-infection DENV antibody profiles: DENV-naïve (pre-infection PRNT_50_<10 for all DENV serotypes), DENV-monotypic (pre-infection PRNT_50_ ≥10 for a single DENV serotype), and DENV-multitypic (pre-infection PRNT_50_ ≥10 for two or more DENV serotypes). To attempt to discern cross-reactive immunity from serotype-specific immunity, we further stratified DENV-multitypics according to mean age of infection, with the logic that older children would have had more time to experience multiple DENV infections and generate multiple serotype-specific responses, while younger children with multitypic profiles would, on average, reflect more cross-reactivity. Preexisting JEV immunity was dichotomized as JEV-positive (pre-infection PRNT_50_≥10) and JEV-negative (pre-infection PRNT_50_<10).

### Inferring the Infecting Serotype

By definition, acute phase blood samples were not available from children with asymptomatic DENV infections for direct detection of the infecting virus. Approximately one-quarter of symptomatically infected individuals were RT-PCR negative, likely because the acute specimen was drawn when the child was no longer viremic. Based upon evidence that DENV transmission in this community is highly clustered, we assumed that the serotypes detected among RT-PCR positive cases attending a given school during a given epidemic year were representative of the serotypes causing asymptomatic and RT-PCR negative symptomatic infections at that school [33], [34]. To minimize the possible biases inherent to this assumption, imputation of an individual's unknown infecting serotype using community-level data was performed only for children residing in communities that had a single serotype detected during that epidemic year. If more than one serotype was detected at a given school during a given year, the infecting serotype was left as missing.

### JEVAX – Description and Administration

The JEV vaccine used in Thailand and throughout Southeast Asia, JEVAX, is a formalin-inactivated, mouse brain-derived JEV vaccine. The first dose of JEVAX in Thailand is administered concurrently with the diphtheria/tetanus/pertussis vaccine at 18 months of age. A second dose is administered 1 to 4 weeks later and a third (booster) dose is administered at 30 to 36 months of age. The seroconversion rates with JEVAX in Thai children have been estimated to be 50% following primary vaccination, 64–80% following secondary vaccination, and 100% following receipt of the third dose [35]. Based upon these observations of suboptimal seroconversion rates with the two dose regimen, the third dose was added to the Expanded Program for Immunization (EPI) regimen in Thailand in 2000.

### History of JEV Vaccination Programs in Kamphaeng Phet

A Phase III trial of JEVAX was conducted in 1984 in Kamphaeng Phet, Thailand, which demonstrated favorable efficacy [8]. JEVAX began to be slowly incorporated into the EPI in Thailand in 1988 and became part of Kamphaeng Phet's EPI in 1992. Vaccination histories for the cohort study were obtained by self-report and subsequent verification with clinic or family documents was not possible. However, given the ages of the children enrolled (five to fourteen years of age) and the timing of the study (1998 to 2002), it is likely that the majority of children enrolled in the cohort study would have received two doses of JEV vaccine. Younger children would likely have been vaccinated as infants, as part of the EPI program, and older children would likely have been vaccinated during the large “catch-up" vaccination programs taking place in the province in the 1990s. The JEVAX vaccine strain in use during the 1990s in Thailand was the Nakayama strain, the country has since switched to the Beijing strain.

### Statistical Analyses

Bivariate analyses were performed using chi-square testing for categorical variables and ANOVA or nonparametric testing for continuous variables. Logistic regression models for symptomatic versus asymptomatic infection were constructed using SAS' GENMOD procedure, accounting for the clustering of observations by school. The best model was then chosen by backward regression. Analyses were performed using SAS software, version 8 (SAS Institute, Cary, NC), SPSS for Windows version 10.0 (SPSS Inc., Chicago, IL), and R version 2.10.1 (R Foundation for Statistical Computing, Vienna, Austria).

## Results

A total of 942 children experienced at least one DENV infection between 1998 and 2002, out of 3,687 children who were enrolled for at least one full year. While some experienced multiple DENV infections during their enrollment, analyses are restricted to the first detected infection for each child. Further characterization of infections as symptomatic or asymptomatic was limited to those 569 cases that occurred within the active surveillance period (June 1 – November 1). Three-hundred-sixteen (56%) of these infections were asymptomatic and 253 (44%) were symptomatic. Of the symptomatic cases, 217 (86%) were DF and 36 (14%) were DHF (Table 1). No deaths were attributed to DENV infection.

**Table 1:** Cohort characteristics associated with symptomatic DENV illness.

	# Infections *(% of Total)	% Symptomatic	p-value **
Total infections	569	44.5%	
Infection Severity			
Asymptomatic	316 (55.5%)	–	
DF	217 (38.1%)	–	–
DHF	36 (6.3%)	–	
JEV NAb Status			
Negative	265 (46.6%)	46.0%	
Positive	214 (37.6%)	57.0%	0.021
Missing	90 (15.8%)	10.0%	
Age (years)			
7-9	216 (38.0%)	47.7%	
10-12	341 (59.9%)	42.2%	0.423
13-15	10 (1.8%)	50.0%	
Missing	2 (0.4%)	50.0%	
Gender			
Male	275 (48.3%)	42.5%	
Female	292 (51.3%)	46.2%	0.699
Missing	2 (0.4%)	50.0%	
School (12 total)			
Range (Min – Max)	5–139	32.3–80.0%	0.618
Epidemic Year			
1998	168 (29.5%)	42.3%	
1999	133 (23.4%)	42.9%	
2000	46 (8.1%)	26.1%	0.032
2001	173 (30.4%)	51.4%	
2002	49 (8.6%)	49.0%	
Pre-Infection DENV NAb Profile			
DENV-Naïve	83 (14.6%)	55.4%	
Monotypic	74 (13.0%)	59.5%	0.131
Multitypic	322 (56.6%)	47.8%	
Missing	90 (15.8%)	10.0%	
Reported JEV Vaccination			
Yes	311 (54.7%)	40.8%	
Can't recall	108 (19.0%)	51.9%	0..119
No	149 (26.1%)	36.3%	
Missing	1 (0.2%)	0.0%	

*: Refers to first-detected DENV infections in the cohort that occurred during the active surveillance period each year (June 1–November 1) and had neutralizing antibody data available

**: Statistical tests considered the association between variables of interest and the occurrence of symptomatic versus asymptomatic infection. p-values were obtained using the Pearson chi-square test for categorical variables, with α = 0.05 as the level of significance. ‘Missing’ categories were not included in statistical comparisons.

### Factors Associated with Symptomatic Illness

Children with JEV NAbs were more likely to experience symptomatic infection than children without JEV NAbs (57% versus 46%, p = 0.021 by χ^2^ testing, Table 1). Of the 569 first detected DENV infections, 479 had pre-infection DENV and JEV neutralizing antibody titer information available for analysis (84.2%). 90% of the missing NAb data were associated with asymptomatic infections, for reasons described above.

There were no differences by age, gender, or school in the proportions of infections that were symptomatic. The proportion of infections that were symptomatic varied by year, from 26% in 2000 to 51% in 2001 (p = 0.032, by χ^2^ testing across all strata of epidemic year). There were no differences in the proportion symptomatic by pre-infection DENV antibody status. There was no difference in the proportion symptomatic by reported JEV vaccination history and, among those reporting a history of vaccination, no difference in the time between vaccination and the first detected infection (mean±SD 5.01±2.22 years for asymptomatics, 4.93±2.11 for symptomatics).

### Factors Associated with JEV Positivity

Children developing DF were most likely to be JEV NAb positive, those experiencing asymptomatic infection the least (50% versus 39%, p = 0.017, by χ^2^ testing across all strata of infection severities) (Table 2). There were no differences in the proportions of children that were JEV NAb positive by age or gender. The proportion JEV NAb positive varied by school and year. DENV-naives were most likely to be JEV NAb positive (54%), then DENV-multitypics (47%), then DENV-monotypics (26%) (p = 0.001 by χ^2^ testing). Those reporting a history of JEV vaccination were more likely to be JEV NAb positive (46% versus 31% p = 0.012 by χ^2^ testing). Among those reporting a history of vaccination, there was no difference in time since vaccination (mean±SD 5.01±2.27 years for JEV NAb positives, 4.96±2.19 for JEV NAb negatives).

**Table 2:** Factors associated with JEV seropositivity among those experiencing dengue (DENV) infection.

	# Infections * (% of Total)	% JEV positive	p-value **
Total DENV infections	479	44.7%	
DENV Infection Severity			
Asymptomatic	235 (49.1%)	39.1%	
DF	211 (44.1%)	50.2%	0.017
DHF	33 (6.9%)	48.5%	
Age (years)			
7–9	185 (38.6%)	43.2%	
10–12	285 (59.5%)	44.9%	0.209
13–15	8 (1.7%)	75.0%	
Missing	1 (0.2%)	100.0%	
Gender			
Male	232 (48.4%)	44.8%	
Female	246 (51.4%)	44.7%	0.667
Missing	1 (0.2%)	100.0%	
School (12 total)			
Range (Min – Max)	4 – 127	13.3% – 75.0%	0.004
Dengue Epidemic Year			
1998	145 (30.3%)	36.6%	
1999	104 (21.7%)	57.7%	
2000	32 (6.7%)	50.0%	0.021
2001	159 (33.2%)	42.1%	
2002	39 (8.1%)	46.2%	
Pre-Infection DENV NAb Profile			
DENV-Naïve	83 (17.3%)	54.2%	
Monotypic	74 (15.4%)	25.7%	0.001
Multitypic	322 (67.2%)	46.6%	
Reported JEV Vaccination			
Yes	259 (54.1%)	51.0%	
Can't recall	95 (19.8%)	38.9%	0.012
No	124 (25.9%)	36.3%	
Missing	1 (0.2%)	0.0%	

*: Refers to first-detected DENV infections in the cohort that occurred during the active surveillance period each year (June 1 – November 1) and had neutralizing antibody data available.

**: Statistical tests considered the association between variables of interest and the presence or absence of JEV NAbs in the pre-infection sample. p-values were obtained using the Pearson chi-square test for categorical variables, with α = 0.05 as the level of significance. ‘Missing’ categories were not included in statistical comparisons.

### Associations of JEV NAb Positivity with DENV Illness

JEV NAb positivity was associated with an increase in the odds of symptomatic infection in unadjusted analysis (OR = 1.55, 95% CI: 1.08 to 2.23) (Table 3). There were no significant differences in the occurrence of hospitalized illness or the occurrence of DHF.

**Table 3:** Unadjusted associations of DENV illness severity and JEV antibody status.

	# Infections	% JEV Positive	OR (95% CI) †
Symptomatic	244	50.0%	1.55 (1.08–2.23)
Asymptomatic	235	39.1%	
Hospitalized	48	43.8%	0.96 (0.53–1.75)
Non-hospitalized *	431	44.8%	
DHF	33	48.5%	1.18 (0.58–2.39)
Non-DHF **	446	44.4%	

*: Non-hospitalized infections incorporate both non-hospitalized DF and asymptomatic seroconversions.

**: Non-DHF infections incorporate both non-hospitalized and hospitalized DF and asymptomatic seroconversions.

**†:** P-values were calculated using the Mantel-Haenzel chi-square statistic.

**Figure 1:**
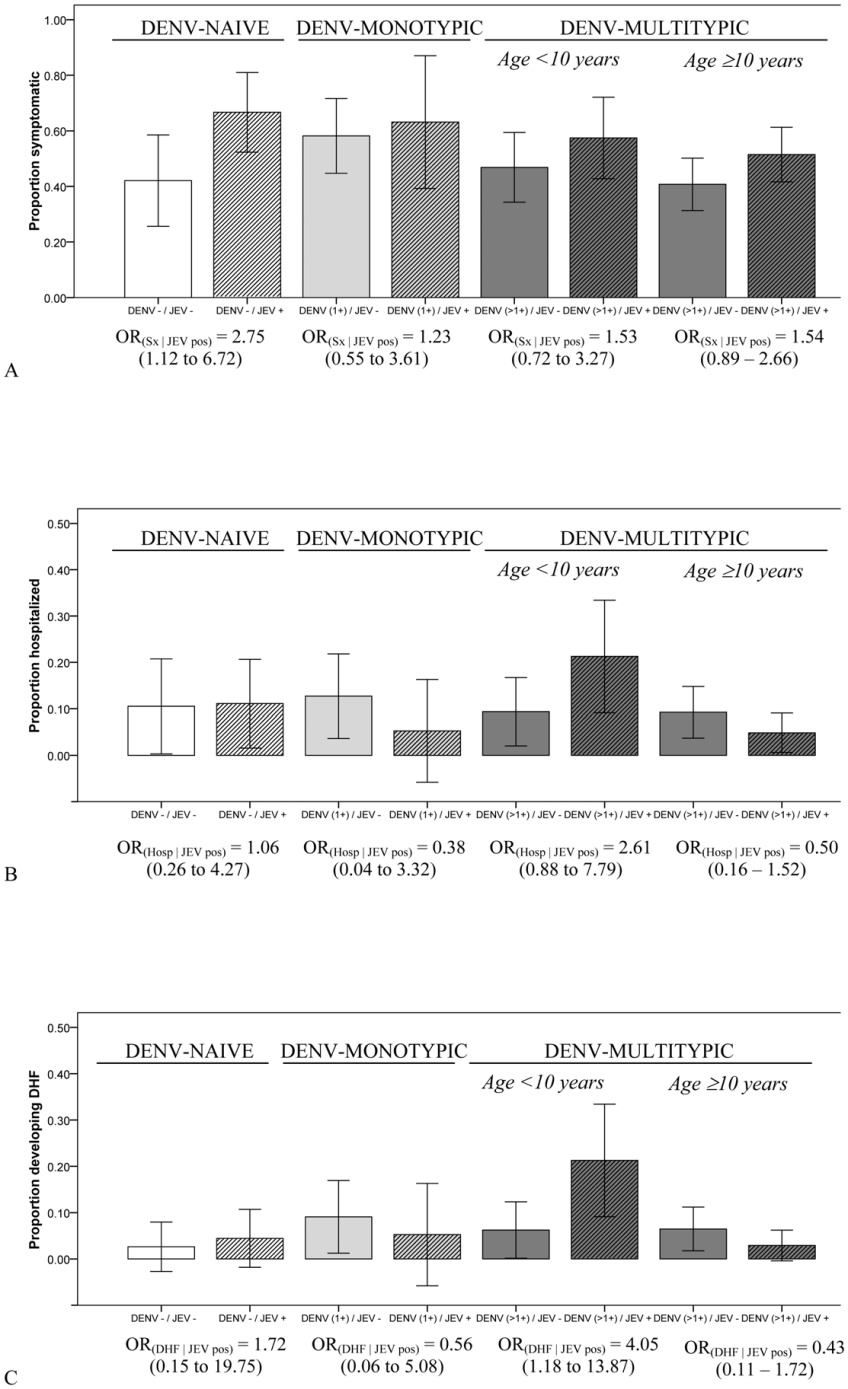
Clinical severity of dengue infections by strata of preexisting DENV and JEV immunity. The proportions of dengue (DENV) infections resulting in symptomatic illness (1a), hospitalized illness (1b), and dengue hemorrhagic fever (1c) are shown. Data are stratified by preexisting DENV immunity (naïve [DENV -], monotypic [DENV 1+], multitypic older and younger than 10 years of age [DENV >1+]) and preexisting Japanese encephalitis virus (JEV) neutralizing antibodies (NAbs) (positive [+] or negative [-]). The JEV NAb positive groups are indicated by hash lines for each stratum of DENV immunity. Odds ratios (ORs) estimate the odds of being experiencing the disease severity of interest (dengue hemorrhagic fever [DHF], hospitalized illness [Hosp], or symptomatic illness [Sx]) in the presence of JEV NAbs over the odds of experiencing the disease severity of interest in the absence of JEV NAbs. Values in parentheses indicate the 95% confidence intervals for the ORs. Error bars indicate the 95% confidence intervals for proportions.

Individuals with JEV NAbs were more likely to experience symptomatic DENV infection for all strata of preexisting DENV immunity, though this association was strongest and significant only for DENV-naives (OR = 2.75, 95% CI: 1.12 to 6.72) (Figure 1a). The association was non-significant and weakest for children with DENV-monotypic immunity prior to infection. Younger and older children with DENV-multitypic immunity had approximately the same increased odds of symptomatic infection with JEV NAbs; neither association was significant.

The directions of the associations between JEV NAbs and hospitalized illness (Figure 1b) and JEV NAbs and DHF (Figure 1c) were not consistent across strata of preexisting DENV immunity and the associations were largely non-significant. The association between JEV NAbs and DHF was significant only for younger DENV-multitypics, who had increased odds of DHF with JEV NAbs (OR = 4.05, 95% CI: 1.18 to 13.87).

### Duration of Illness

The presence of JEV NAbs was associated with an increased duration of DENV illness in DENV-naives (5.70 versus 2.69 days, p = 0.045) (Table 4). For DENV-monotypics and multitypics, no difference in the duration of illness was observed.

**Table 4:** Duration of DENV illness (days) by JEV NAb status.

Mean duration of illness in days (SD) *	JEV NAb -	JEV NAb+	p-value **
All symptomatic infections	4.51 (5.28)	4.89 (5.66)	0.591
DENV-Naives	2.69 (1.74)	5.70 (5.68)	0.045
DENV-Monotypic	5.52 (7.62)	4.75 (5.79)	0.756
DENV-Multitypic	4.51 (4.58)	4.60 (5.68)	0.915

*: Duration of illness was calculated as the number of days elapsed from first day of febrile illness to the last day that a child reported any fever, muscle or joint pain, headache, nauseas, vomiting, diarrhea, or any signs of bleeding or hemorrhage.

**: P-values were calculated using 2-way analysis of variance testing (ANOVA), with α = 0.05 as the level of significance.

### Titer of JEV NAbs

**Figure 2:**
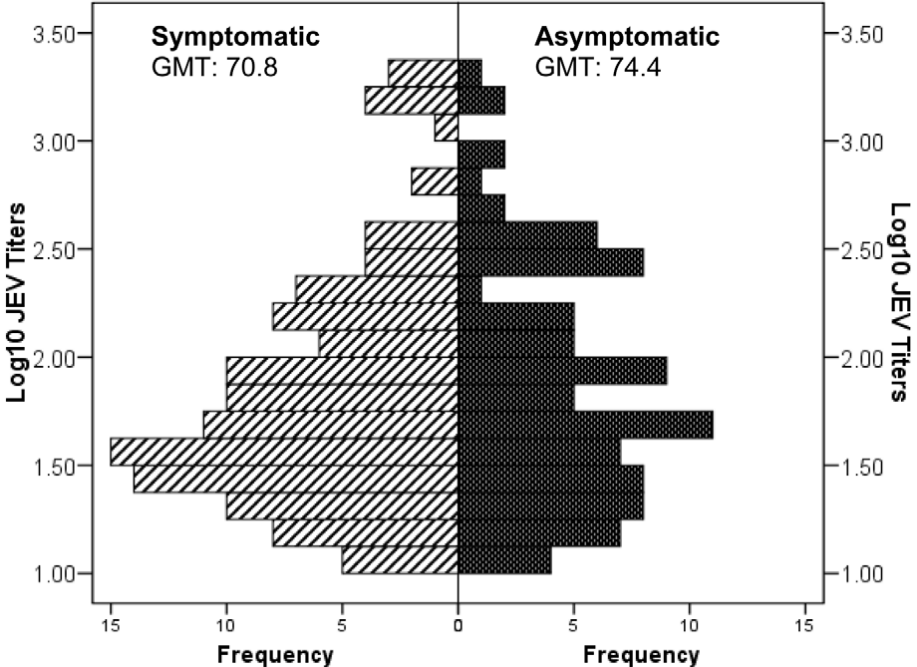
Distribution of JEV neutralizing antibody titers among those with detectable JEV antibodies. Log10 titers are shown for asymptomatic (dark gray) and symptomatic (striped) infections. JEV: Japanese encephalitis.

Among those with JEV NAbs prior to infection, there was no difference in the geometric mean titer between asymptomatically and symptomatically infected individuals (p = 0.45 by Mann-Whitney U test, Figure 2). The distributions of the titers were similar.

### Role of Infecting DENV Serotype

**Figure 3:**
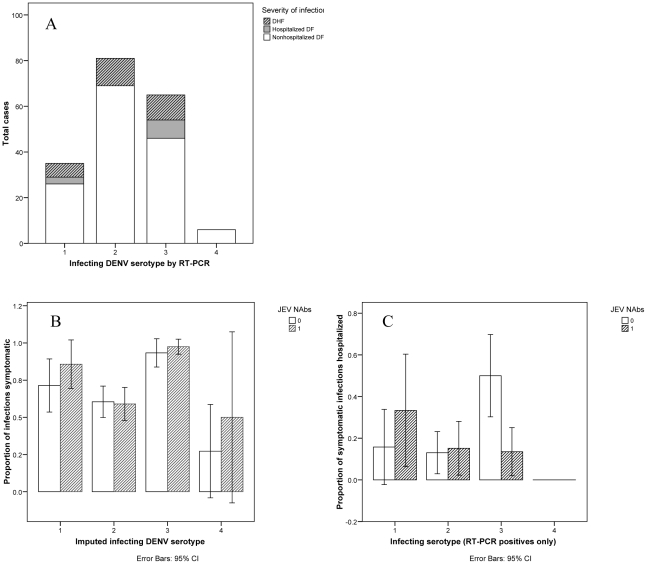
Associations between JEV neutralizing antibodies and clinical severity of DENV infections, by DENV serotype. The total number of cases of non-hospitalized dengue fever (DF), hospitalized DF, and dengue hemorrhagic fever (DHF) are shown by dengue (DENV) serotype (3a). The proportions of infections developing symptomatic illness (3b) and hospitalized illness (3c) are shown by Japanese encephalitis (JEV) neutralizing antibody (NAb) status and infecting serotype. JEV NAb positive groups are represented by hash lines; JEV NAb negatives by white bars. For figure 3b, the infecting DENV serotype was imputed for asymptomatic and RT-PCR-negative symptomatic infections, using information on the predominate DENV serotype in circulation at a child's school for the time interval during which they were infected, and restricting to those schools that had only one serotype in circulation that year (as detected by RT-PCR). Analysis was restricted to RT-PCR-positive, and therefore symptomatic, infections in figure 3c. Error bars indicate the 95% confidence interval for the proportion of children developing symptomatic illness (3b) and hospitalized illness (3c).

The greatest number of RT-PCR-positive illnesses in the cohort was associated with DENV-2; the highest proportion of hospitalized infections was observed with DENV-3 (Figure 3a). The infecting DENV serotype was unknown for all asymptomatic infections and for 25.2% of symptomatic infections. 40.0% of children missing serotype data resided in communities with a single serotype in circulation the year of their infection and were therefore eligible for imputation. The probability of symptomatic infection was increased with JEV NAbs for all DENV serotypes, but the association was not significant for any serotype in subgroup analysis (Figure 3b). Limiting the comparisons to symptomatic, RT-PCR positive infections (with no imputation), the direction of the association between JEV NAbs and hospitalized illness was highly variable (Figure 3c). DENV-3 infection in the setting of preexisting JEV NAbs was associated with decreased hospitalized illness (15.0% hospitalized with JEV NAbs versus 46.7% hospitalized without, p = 0.004 by χ2 test).

### Multivariate Model of JEV NAb Positivity and Symptomatic DENV Infection

The positive, significant association between the presence of JEV NAbs and symptomatic infection remained after controlling for age, pre-infection DENV immunity, and epidemic year and accounting for clustering of observations by school (data not shown). The adjusted odds ratio was 1.70 (95% CI 1.15 to 2.51).

## Discussion

The serological cross-reactivity between DENV serotypes is well-documented and has been linked to both cross-protection and enhanced disease. In contrast, the clinical implications of serological cross-reactivity observed between DENV and other non-DENV flaviviruses remain unclear. In this study, we characterized the association between preexisting JEV antibodies and the clinical severity of subsequent DENV infection in a prospective study of school-children in Thailand. We report the novel finding that JEV NAbs were associated with the increased occurrence of symptomatic DENV infection.

The increased occurrence of symptomatic illness with preexisting JEV NAbs was most pronounced in children who were DENV-naïve (i.e., presumably experiencing their first DENV infection), for whom JEV NAbs were also associated with a longer duration of illness. It is important to note that given the sensitive method of identifying febrile illnesses in this cohort, ‘symptomatic DENV illnesses’ ranged from a single day of fever to a prolonged and debilitating disease course. It is therefore notable that we observed an increase in the duration of illness in those with JEV NAbs, suggesting that their influence was to increase the occurrence and severity of clinically-meaningful DENV illness. The significant findings in DENV-naïve children are noteworthy because in this group, JEV NAbs are more likely to reflect a ‘true’ prior exposure to JEV or JEV vaccine in this group and less likely to have arisen as a cross reactive response to a prior DENV infection. The association between JEV NAbs and symptomatic illness was not as strong for DENV-monotypics and -multitypics, which could be due to cross-protection with increasing DENV immunity and/or a confounding effect with the inclusion of JEV NAbs as a result of cross-reactivity.

Given the absence of confirmatory data from other human cohorts or animal models, we must place these findings in the context of prior in vitro investigations. This study may be most analogous and in accordance with Putvatana et al, who found no evidence of antibody-dependent enhancement of DENV-2 infection in vitro using sera of JEV-immune Thai individuals [21]. We report an increase in non-hospitalized DENV illnesses with preexisting JEV NAbs, but not DHF (in unadjusted analysis and for nearly all subgroups), which may suggest that enhancement is less likely to be the mechanism of increased DENV illness. However, the number of DHF cases was low in this cohort study and therefore the power to detect an association between JEV antibodies and severe DENV illness was limited. It is also possible that studies of this association, both in vivo and in vitro, could have different conclusions based upon the serotypes and strains under consideration [18]. Indeed, while only limited serotype-specific analyses were possible with these data, the nature of the association between JEV NAbs and hospitalized illness did not appear to be consistent across serotypes. Finally, one important caveat with this in vivo study is that while an association was indeed detected between JEV NAbs and DENV illness, it is possible that JEV NAbs are in fact a marker of another underlying biological function that was not considered in this analysis, such as cell-mediated immunity.

Notably, there was not a strong independent association in this study between preexisting DENV immunity and the clinical severity of a subsequent DENV infection; this may at first glance appear to be in contrast to prior studies linking secondary DENV infection with an increased occurrence of hospitalized illness or DHF [36], [37]. However, the present study uniquely focused on predictors of symptomatic (primarily non-hospitalized) infection, for which the influence of prior DENV exposures and DENV immunity may be different. Second, while DENV-naives and DENV-monotypics may be somewhat reliably characterized as having no prior DENV infections and one prior DENV infection, respectively, the DENV-multitypic group likely comprises a mixture of children with multiple prior DENV infections as well as children with a single prior DENV infection and a persistent cross-reactive response. This ‘mixing of effects’ within the DENV-multitypic group may have clouded the association between DENV immunity and the clinical severity of DENV infection.

It is possible that some children were misclassified with respect to pre-infection JEV serostatus in this study. The prevalence of JEV NAbs in children experiencing DENV infection in the cohort was 45%, remarkably low given that vaccine coverage was estimated to exceed 80% at the time of the study. This low seropositivity is likely due to waning of the antibody response, which is a well-documented phenomenon with JEV inactivated vaccines and particularly with the two-dose regimen [35], [38]. 60% of children who lacked JEV NAbs ‘seroconverted’ to become JEV NAb positive in the post-season sample following a DENV infection, which may reflect an anamnestic response to prior JEV vaccination. Additionally, 76% of DENV-naïve/JEV-negatives exhibited a secondary-type response by ELISA during acute infection, further suggesting that negative JEV and DENV antibody titers do not preclude the possibility of a prior flavivirus infection or JEV vaccination.

It is conceivable that different sources of JEV NAbs, which could not be discerned in this study, may modulate the severity of DENV infection in different ways. Given the cross-reactivity between JEV and DENV in serological assays, it is possible that JEV NAbs in some cases were present as a cross-reactive response to a prior DENV infection. Further, while JEV vaccination was widespread during the study period, wild-type JEV continued to circulate and cause human infections. In summary, the JEV antibodies detected in the cohort may have arisen as a result of JEV vaccination, JEV infection, cross-reactivity from DENV infection, or combinations of these. Future studies should seek to distinguish between vaccine-derived JEV immunity and immunity derived from natural exposure, perhaps by analysis of NS1-specific antibodies [39].

The identification of asymptomatic DENV seroconversions using sequential HI antibody data is a unique strength of this study. However, it should be noted that the sensitivity of this method to detect post-primary DENV infections, or DENV infections in JEV immunes, has not yet been validated against a gold standard. It is therefore possible that some DENV infections were missed or misclassified as JEV infections (i.e., false negatives), or that assay and/or biological variability caused an elevation of HI titers where no new infection had in fact occurred (i.e., false positives).

This first report of an association between preexisting JEV NAbs and DENV illness warrants further study. Because this study was conducted on a relatively limited temporal, spatial, and demographic scale, similar analyses should be repeated in other cohorts. It would be of particular interest to compare DENV-endemic regions where live-attenuated and inactivated JEV vaccines are in use. Possible associations between other flaviviruses and DENV illness should be evaluated, and possible effect-modifying effects of the infecting DENV serotype, should be explored. It would be of great interest to investigate the association between JEV cell-mediated immunity induced by JEV vaccination and wild-type infection and the occurrence of DENV illness.

The biological mechanism of this association remains to be elucidated. However, an intriguing mechanistic possibility may be found in the literature surrounding other inactivated virus vaccines. Some inactivated vaccines have been linked with increased disease, perhaps due to the generation of an epitope-restricted, lower titer immune response. Early efforts to develop inactivated vaccines against measles and respiratory syncytial virus were abandoned after they were linked with the occurrence of atypical, occasionally severe disease [40], [41] There have been no reports to date of immuno-pathological responses following inactivated flavivirus vaccination in humans, though a recent study in mice reported that low doses of JEVAX were associated with increased viral load and death following subsequent Murray Valley encephalitis virus challenge, relative to placebo, high doses of JEVAX, and the live Chimerivax-JEV vaccine [42].

In summary, we report that the prior existence of JEV NAbs was associated with an increased probability of symptomatic DENV illness in a cohort of school-children in Thailand. These findings have public health importance in that DENVs co-circulate with other flaviviruses in much of their geographic range (e.g., JEV in Asia, yellow fever virus in Africa and South America, and West Nile virus in various locations in both hemispheres) and JEV vaccination is common throughout South and Southeast Asia. We suggest that the issue of heterologous flavivirus immunity and DENV, usually considered to be inconsequential or perhaps protective, merits renewed interest and investigation. In particular, the findings indicate that DENV vaccine developers should include preexisting flavivirus immunity and vaccination histories in assessments of vaccine safety and efficacy. The results of these studies may be important for shaping DENV vaccine implementation strategies.

## References

[1] Grossman RA, Gould D, Smith TJ, Johnsen DO, Pantuwatana S (1973). Study of Japanese encephalitis virus in Chiangmai valley, Thailand. I. Introduction and study design.. Am J Epidemiol.

[2] Endy TP, Nisalak A (2002). Japanese encephalitis virus: ecology and epidemiology.. Curr Top Microbiol Immunol.

[3] Chunsuttiwat S (1998). Issues related to integration of JE vaccine into national EPI: experience from Thailand..

[4] Martin DA, Biggerstaff BJ, Allen B, Johnson AJ, Lanciotti RS (2002). Use of immunoglobulin m cross-reactions in differential diagnosis of human flaviviral encephalitis infections in the United States.. Clin Diagn Lab Immunol.

[5] Makino Y, Tadano M, Saito M, Maneekarn N, Sittisombut N (1994). Studies on serological cross-reaction in sequential flavivirus infections.. Microbiol Immunol.

[6] Sabin AB (1952). Research on dengue during World War II.. Am J Trop Med Hyg.

[7] Halstead SB, Chow J, Marchette NJ (1973). Immunologic enhancement of dengue virus replication.. Nature New Biology.

[8] Hoke CH, Nisalak A, Sangawhipa N, Jatanasen S, Laorakpongse T (1988). Protection against Japanese encephalitis by inactivated vaccines.. N Engl J Med.

[9] Gibbons RV, Kalanarooj S, Jarman RG, Nisalak A, Vaughn DW (2007). Analysis of Repeat Hospital Admissions for Dengue to Estimate the Frequency of Third or Fourth Dengue Infections Resulting in Admissions and Dengue Hemorrhagic Fever, and Serotype Sequences.. Am J Trop Med Hyg.

[10] Kanesa-thasan N, Hernandez L, Lyons A, Putnak R, Sun W

[11] Bhamarapravati N, Yoksan S, Chayaniyayothin T, Angsubphakorn S, Bunyaratvej A, St.George TD, Kay H, Blok J (1986). Dengue virus vaccine development 2. Clinical, immunological and biological response in flavivirus immune and non-immune human volunteers inoculated with dengue 2 (16681) passaged 53 times in primary dog kidney cells..

[12] Eckels KH, Kliks SC, Dubois DR, Wahl LM, Bancroft WH (1985). The association of enhancing antibodies with seroconversion in humans receiving a dengue-2 live-virus vaccine.. J Immunol.

[13] Kanesa-Thasan N, Sun W, Ludwig GV, Rossi C, Putnak JR (2003). Atypical antibody responses in dengue vaccine recipients.. Am J Trop Med Hyg.

[14] Scott RM, Eckels KH, Bancroft WH, Summers PL, McCown JM (1983). Dengue 2 vaccine: dose response in volunteers in relation to yellow fever immune status.. J Infect Dis.

[15] Burke DS, Lorsomrudee W, Leake CJ, Hoke CH, Nisalak A (1985). Fatal outcome in Japanese encephalitis.. Am J Trop Med Hyg.

[16] Hammon WM, Tigertt WD, Sather GE, Berge TO, Meiklejohn G (1958). Epidemiologic studies of concurrent “VERGIN “ epidemics of Japanese B encephalitis and of mumps on Guam,1947-1948, with subsequent observations including dengue, through 1957.. Am J Trop Med Hyg.

[17] Hammon WM (1969). Observations on dengue fever, benign protector and killer: a Dr Jekyll and Mr Hyde.. Am J Trop Med Hyg.

[18] Price WH, Thind IS (1972). The mechanism of cross-protection afforded by dengue virus against West Nile virus in hamsters.. J Hyg Camb.

[19] Halstead SB, O'Rourke EJ (1977). Antibody-enhanced dengue virus infection in primate leukocytes.. Nature.

[20] Halstead SB, Porterfield JS, O'Rourke EJ (1980). Enhancement of dengue virus infection in monocytes by flavivirus antisera.. Am J Trop Med Hyg.

[21] Putvatana R, Yoksan S, Chayayodhin T, Bhamarapravati N, Halstead SB (1984). Absence of dengue 2 infection enhancement in human sera containing Japanese encephalitis antibodies.. Am J Trop Med Hyg.

[22] Tarr GC, Hammon WM (1974). Cross-protection between group B arboviruses: Resistance in mice ot Japanese B encephalitis and St. Louis encephalitis viruses induce by dengue virus immunization.. Infect Immun.

[23] Imam IZ, Hammon WM (1957). Challenge of hamsters with Japanese B, St. Louis and Murray Valley encephalitis viruses after immunization by West Nile infection plus specific vaccine.. J Immunol.

[24] Goverdhan MK, Kulkarni AB, Gupta AK, Tupe CD, Rodrigues JJ (1992). Two-way cross-protection between West Nile and Japanese encephalitis viruses in bonnet macaques.. Acta Virol (Praha).

[25] Nemeth N, Bosco-Lauth A, Bowen R (2009). Cross-protection between West Nile and Japanese encephalitis viruses in red-winged blackbirds (Agelaius phoeniceus).. Avian Dis.

[26] Martina B, Koraka P, van den Doel P, van Amerongen G, Rimmelzwaan G (2008). Immunization with West Nile virus envelope domain III protects mice against lethal infection with homologous and heterologous virus.. Vaccine.

[27] Endy TP, Chunsuttiwat S, Nisalak A, Libraty DH, Green S (2002). Epidemiology of inapparent and symptomatic acute dengue virus infection: a prospective study of primary school children in Kamphaeng Phet, Thailand.. Am J Epidemiol.

[28] Endy TP, Nisalak A, Chunsuttiwat S, Libraty DH, Green S (2002). Spatial and temporal circulation of dengue virus serotypes: a prospective study of primary school children in Kamphaeng Phet, Thailand.. Am J Epidemiol.

[29] Nimmannitya S, Gubler DJ, Kuno G (1997). Dengue Hemorrhagic Fever: Diagnosis and Management.. Dengue and dengue hemorrhagic fever.

[30] Clarke DH, Casals J (1958). Techniques for hemagglutination and hemagglutination inhibition with arthropod-borne viruses.. Am J Trop Med Hyg.

[31] Russell PK, Nisalak A, Sukhavachana P, Vivona S (1967). A plaque reduction test for dengue virus neutralization antibodies.. J Immunol.

[32] Yoksan S, Bhamarapravati N, Halstead SB (1986). Dengue virus vaccine development: Study on biological markers of uncloned dengue 1-4 viruses serially passaged in primary kidney cells..

[33] Jarman RG, Holmes EC, Rodpradit P, Klungthong C, Gibbons RV (2008). Microevolution of Dengue viruses circulating among primary school children in Kamphaeng Phet, Thailand.. J Virol.

[34] Mammen MP, Pimgate C, Koenraadt CJ, Rothman AL, Aldstadt J (2008). Spatial and temporal clustering of dengue virus transmission in Thai villages.. PLoS Med.

[35] Nimmannitya S, Hutamai S, Kalayanarooj S, Rojanasuphot S (1995). A field study on Nakayama and Beijing strains of Japanese encephalitis vaccines.. Southeast Asian J Trop Med Public Health.

[36] Halstead SB, Nimmannitya S, Cohen SN (1970). Observations related to pathogenesis of dengue hemorrhagic fever. IV. Relation of disease severity to antibody response and virus recovered.. Yale J Biol Med.

[37] Sangkawibha N, Rojanasuphot S, Ahandrik S, Viriyapongse S, Jatanasen S (1984). Risk factors in dengue shock syndrome: a prospective epidemiologic study in Rayong, Thailand. I. The 1980 outbreak.. Am J Epidemiol.

[38] Rodrigues FM, Mohan Rao CV, Mandke VB, Pinto BD, Pavri K (1986). Neutralizing antibody response to Japanese encephalitis inactivated mouse brain vaccine among laboratory personnel.. Trans R Soc Trop Med Hyg.

[39] Shu PY, Chen LK, Chang SF, Yueh YY, Chow L (2001). Antibody to the nonstructural protein NS1 of Japanese encephalitis virus: potential application of mAb-based indirect ELISA to differentiate infection from vaccination.. Vaccine.

[40] Rauh L, Schmidt R (1965). Measles immunization with killed virus vaccine. Serum antibody titers and experience with exposure to measles epidemic.. American Journal of Diseases of Children.

[41] Kim HW, Arrobio JO, Brandt CD, Jeffries BC, Pyles G (1973). Epidemiology of respiratory syncytial virus infection in Washington, D.C. I. Importance of the virus in different respiratory tract disease syndromes and temporal distribution of infection.. Am J Epidemiol.

[42] Lobigs M, Larena M, Alsharifi M, Lee E, Pavy M (2009). Live chimeric and inactivated Japanese encephalitis virus vaccines differ in their cross-protective values against Murray Valley encephalitis virus.. Journal of Virology.

